# Life Table Parameters of Three Mirid Bug (*Adelphocoris*) Species (Hemiptera: Miridae) under Contrasted Relative Humidity Regimes

**DOI:** 10.1371/journal.pone.0115878

**Published:** 2014-12-26

**Authors:** Hongsheng Pan, Bing Liu, Yanhui Lu, Nicolas Desneux

**Affiliations:** 1 State Key Laboratory for Biology of Plant Diseases and Insect Pests, Institute of Plant Protection, Chinese Academy of Agricultural Sciences, Beijing, China; 2 French National Institute for Agricultural Research (INRA), Sophia-Antipolis, France; Universidad Nacional Autonoma de Mexico, Mexico

## Abstract

The genus *Adelphocoris* (Hemiptera: Miridae) is a group of important insect pests of Bt cotton in China. The three dominant species are *A. lineolatus*, *A. suturalis*, and *A. fasciaticollis*, and these species have different population dynamics. The causal factors for the differences in population dynamics have not been determined; one hypothesis is that humidity may be important for the growth of *Adelphocoris* populations. In the laboratory, the demographic parameters of the three *Adelphocoris* species were compared when the mirid bugs were subjected to various levels of relative humidity (40, 50, 60, 70 and 80% RH). Middle to high levels of RH (60, 70 and 80%) were associated with higher egg and nymph survival rates and increased adult longevity and female fecundity. Lower humidity levels (40 and 50% RH) had negative effects on the survival of nymphs, adult longevity and fecundity. The intrinsic rate of increase (*r_m_*), the net reproductive rate (*R_0_*) and the finite rate of increase (*λ*) for each *Adelphocoris* species increased with increasing RH. Significant positive relationships were found between RH and the life table parameters, *r_m_*, *R_0_* and *λ* for the three *Adelphocoris* species. These results will help to better understand the phenology of the three *Adelphocoris* species, and the information can be used in population growth models to optimize pest forecasting and management strategies for these key pests.

## Introduction

The genus *Adelphocoris* (Hemiptera: Miridae) includes three key pest species of cotton (and various other agricultural crops) in China: *Adelphocoris lineolatus* Goeze, *Adelphocoris suturalis* Jakovlev and *Adelphocoris fasciaticollis* Reuter [1], [2], [3]. In past decades, the *Adelphocoris* spp. were considered secondary insect pests in the cotton fields of China, and the mirid bugs were typically controlled with broad-spectrum insecticides used for management of the cotton bollworm *Helicoverpa armigera* (Hübner) [4], [5]. After the widespread adoption of Bt cotton in the late 1990s and the associated decrease in insecticide use in cotton fields [6], [7], mirid bugs (including *Adelphocoris* spp. and *Apolygus lucorum* (Meyer-Dür)) emerged as economically important insect pests of cotton; the bugs caused serious yield losses in the Yangtze River and the Yellow River cotton growing regions of China [5], [8], [9].

The three *Adelphocoris* species differ greatly in their geographical distributions and seasonal population dynamics in China [3], [10], [11]. *Adelphocoris suturalis* is generally found in the Yangtze River region and the southern part of the Yellow River region, whereas *A. lineolatus* and *A. fasciaticollis* are primarily confined to the middle and northern parts of the Yellow River region [2], [12]. *Adelphocoris suturalis* causes serious yield losses in cotton, but infestation levels of the other two species are generally relatively low [3]. These differences in phenology may be related to species-specific biological and ecological characteristics and/or adaptation to biotic factors (e.g., host plants) and abiotic conditions (e.g., temperature, ambient humidity and rainfall). Previous studies have partially documented the differences among *Adelphocoris* species for species-specific flight performance, temperature-dependent development and overwintering host plant range [10], [11], [13]. However, other factors, e.g., environmental humidity, may also be important to explain the occurrence and infestation levels of *Adelphocoris* species in the various regions of China.

Ting [14] found that *Adelphocoris* species and *A. lucorum* preferred shady, moist environments, which indicated that rainfall was an important factor in the regulation of their seasonal population dynamics. Many other field surveys of these mirid bugs found a similar phenomenon, in which high rainfall resulted in rapid population increases [1], [2], [8], [15]. Plants often grow more rapidly after heavy rainfall and thus provide more suitable foods (i.e., tender leaves) for mirid bugs, providing one possible reason for rainfall-dependent outbreaks [3], [16], [17]. Another major change after rainfall is a marked increase in the relative humidity (RH). Lu and Wu [18] previously reported that *A. lucorum* had higher individual survival and adult fecundity when reared at 70 and 80% RH than when reared at 40 and 50% RH. Hence, we hypothesized that RH greatly affects the population growth of three *Adelphocoris* species.

In the present study, egg and nymph survival and development, adult longevity and fecundity, and various key life table parameters were compared for the three *Adelphocoris* species reared in five different RH regimes. The results will contribute to our understanding of the causes for the phenology of these three key *Adelphocoris* pest species.

## Materials and Methods

### Ethics Statement

Local farmers gave permission to collect mirid bugs in their fields, and no permits were required to collect these three common insects. The sampling did not involve regulated, endangered or protected species.

### Insect rearing

Laboratory colonies of the three *Adelphocoris* species were established with field-collected individuals. The nymphs and adults of *A. suturalis* were collected from cotton fields in Xinxiang (Henan Province, 35.32°N and 113.85°E) in July and August, whereas nymphs and adults of *A. lineolatus* and *A. fasciaticollis* were collected from alfalfa fields in Cangzhou (Hebei Province, 38.33°N and 116.83°E) and Langfang (Hebei Province, 39.53°N and 116.70°E) during July and August, respectively. Each laboratory colony was initiated with 500–800 field-collected individuals. The mirid bugs were placed in plastic rearing boxes (20×10×6 cm) at the rate of 60–80 bugs per box. The insects were reared on green beans (*Phaseolus vulgaris* L.) and were provided 10% sucrose solution [19]. The green beans also served as the oviposition site and were changed every two days. Beans with eggs were placed in new rearing boxes (with filter paper) and were maintained until first instar nymphs emerged. The nymphs were placed in similar boxes (at the rate of 100 nymphs per box) that were covered with nylon mesh to allow air circulation and provided with fresh green beans every 2 days until adult emergence. The laboratory colonies were maintained at 29±1°C and 60±5% RH with a 14∶10 L:D photoperiod. Mirid bugs of the third generation were used for the experiments.

### Experimental design

The methodology used was similar to that described by Lu et al. [10] and Lu & Wu [18]. Adults of the three mirid bug species were held for 24 h in rearing boxes for oviposition (with green beans as the oviposition substrate). After 24 h, the beans containing eggs were placed in new rearing boxes in growth chambers (Ningbo Jiangnan Instrument Factory, Ningbo, China) at 25±0.5°C with a 14∶10 L:D photoperiod but with various levels of RH. The humidity levels that were assessed were 40, 50, 60, 70 and 80% RH. A total of 65–121 eggs per *Adelphocoris* species were examined at each RH level. Meanwhile, many other eggs per species were maintained under each RH condition to provide enough nymphs for the next trial.

The hatching of eggs was recorded daily, and the newly emerged nymphs were collected and placed individually into glass vials (height 5 cm and diameter 1.5 cm), covered with a nylon screen, provided a small section of green bean and a paper strip (1×5 cm), and then kept at the same RH level. The survival and development of the nymphs were recorded daily for the various RH regimes until either adulthood or death occurred. The number of nymphs per RH level per species ranged from 60 to 120. At the same time, many other individuals per species were kept at different RH levels as above to breed enough adults for the next assay.

Upon emergence, the adults were collected and paired. Each mating pair was placed into a glass vial covered by a nylon screen (height 3 cm and diameter 3 cm). A 2.5-cm section of green bean was provided as food and oviposition substrate, and a 10% sucrose solution was provided as a food supplement (on a cotton ball). The adult mortality was checked daily, and the green bean was replaced daily. The longevity of *Adelphocoris* adults and the fecundity of each mating pair was determined for each of the five RH levels. Each treatment (RH level) was assessed with 45–90 mating pairs per species.

### Data analyses

Statistical analyses were performed with SAS [20]. Egg hatching and nymph survival rates were compared with a chi-square goodness-of-fit test among the various RH levels. The effects of the RH on the development of the immatures, adult longevity and fecundity of each *Adelphocoris* species were analyzed with one-way analysis of variance (ANOVA), and the means were separated by Tukey's honest significant difference (HSD) test (*P* = 0.05). Before ANOVA, the adult fecundity data were log_10_ (n+1) transformed (all data sets were tested for normality prior to ANOVA). Nymphs that died prior to becoming adults and adults that died without producing any eggs were excluded from analysis of nymph development and adult fecundity, respectively.

The following life table parameters were calculated for each species for all RH levels: the net reproductive rate, (*R_0_*) = ∑*l_x_m_x_*; the intrinsic rate of increase (*r_m_*), which was calculated by iteratively solving the equation ∑*l_x_m_x_e^-rmx^* = 1; the mean generation time, (*T*) = ln*R_o_*/*r_m_*; and the finite rate of increase, (*λ*) = *e^rm^*. In the equations, *l_x_* is the age-specific survival rate, which is the probability to survive to a particular age x, and *m_x_* is the age-specific fecundity, which is calculated as the number of live females per female for age x. The Jackknife procedure was used to estimate a standard error for the *r_m_* values at different RH levels. The relationships between RH and life table parameters were characterized by linear regression analysis using the model y = bx+a, where y is the life table parameter, x is RH, and a and b are coefficients obtained from the regression.

## Results

### Effect of humidity on survival and development of nymphs

**Figure 1:**
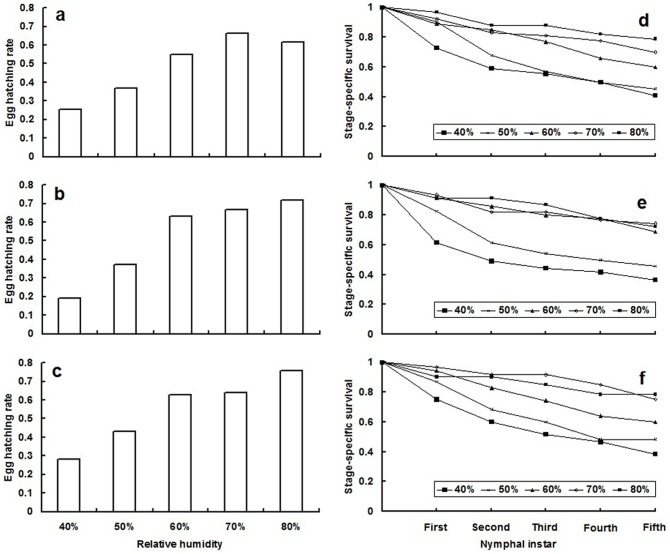
Stage-specific survival of eggs and nymphs of three *Adelphocoris* species at different levels of relative humidity (RH). (a) and (b) are eggs and nymphs of *A. lineolatus*, respectively; (c) and (d) are eggs and nymphs of *A. suturalis*, respectively; and (e) and (f) are eggs and nymphs of *A. fasciaticollis*, respectively.

Relative humidity significantly affected the survival of eggs and nymphs of the three *Adelphocoris* species (*A. lineolatus*: eggs, χ^2^ = 41.25, df = 4, *P*<0.001; nymphs, χ^2^ = 40.59, df = 4, *P*<0.001; *A. suturalis*: eggs, χ^2^ = 84.28, df = 4, *P*<0.001; nymphs, χ^2^ = 51.31, df = 4, *P*<0.001; and *A. fasciaticollis*: eggs, χ^2^ = 45.94, df = 4, *P*<0.001; nymphs, χ^2^ = 29.17, df = 4, *P*<0.001). The eggs and nymphs of all three *Adelphocoris* species had the lowest survival at 40% RH. The highest egg hatch rate of *A. suturalis* and *A. fasciaticollis* was at 80% RH and that of *A. lineolatus* was at 70%. The highest survival of *A. lineolatus*, *A. suturalis*, and *A. fasciaticollis* nymphs was at 60%, 70% and 80% RH, respectively (Fig. 1). The RH level significantly affected the development times of *A. lineolatus* nymphs (*F*_4,275_ = 5.80, *P*<0.001) and *A. suturalis* eggs (*F*_4,230_ = 2.70, *P* = 0.031) and nymphs (*F*_4,288_ = 5.52, *P*<0.001), but the development times of the eggs of *A. lineolatus* and *A. fasciaticollis* and the nymphs of *A. fasciaticollis* were not affected (*A. lineolatus* eggs: *F*_4,215_ = 1.55, *P* = 0.190; *A. fasciaticollis* eggs: *F*_4,209_ = 2.20, *P* = 0.070; and *A. fasciaticollis* nymphs, *F*_4,181_ = 1.82, *P* = 0.126, see Table 1).

**Table 1:** Effect of relative humidity on the development of immatures, adult longevity and fecundity of three *Adelphocoris* species.

Species	Relative humidity (%)	Development duration (d)		Longevity (d)		
		Egg	Nymph	Female	Male	Fecundity (eggs per female)
*Adelphocoris lineolatus*	40	10.67±0.23 a	16.71±0.19 a	19.26±1.12 c	13.45±1.20 c	25.58±3.40 c
	50	10.43±0.21 a	16.02±0.21 b	25.10±1.15 bc	22.19±1.82 bc	32.94±4.16 bc
	60	10.64±0.12 a	15.50±0.15 b	38.10±3.57 ab	31.50±2.87 ab	40.75±4.41 abc
	70	10.85±0.15 a	16.05±0.13 b	42.88±5.11 a	38.06±4.53 a	57.48±6.09 a
	80	10.96±0.14 a	16.17±0.18 a	43.51±5.17 a	34.65±4.54 ab	47.86±5.68 ab
*Adelphocoris suturalis*	40	10.91±0.15 ab	16.30±0.18 b	19.71±1.35 c	13.35±1.28 b	13.35±2.32 b
	50	10.54±0.10 b	16.05±0.26 b	27.55±1.76 bc	25.57±2.18 ab	19.88±2.24 b
	60	11.05±0.12 a	16.61±0.20 ab	38.85±3.94 ab	34.03±3.99 a	43.18±6.17 a
	70	11.09±0.10 a	15.99±0.20 b	46.79±5.21 a	39.96±6.05 a	58.33±6.25 a
	80	10.89±0.13 ab	17.28±0.28 a	43.90±5.63 a	39.65±5.36 a	51.35±5.62 a
*Adelphocoris fasciaticollis*	40	11.30±0.25 a	17.17±0.31 a	17.73±1.58 b	17.55±3.33 a	12.09±3.24 c
	50	12.05±0.22 a	17.00±0.47 a	26.10±3.28 b	23.60±5.02 a	21.20±3.66 bc
	60	11.25±0.27 a	16.24±0.25 a	34.60±2.17 ab	24.20±2.81 a	27.40±4.83 bc
	70	11.17±0.23 a	16.18±0.17 a	46.08±6.19 a	34.69±8.12 a	38.85±5.56 ab
	80	11.24±0.20 a	16.53±0.32 a	45.91±6.65 a	31.36±5.89 a	53.36±6.84 a

Means (±SEM) within a column followed by different letters are significantly different (*P*<0.05).

### Effect of humidity on longevity and fecundity of adults

**Figure 2:**
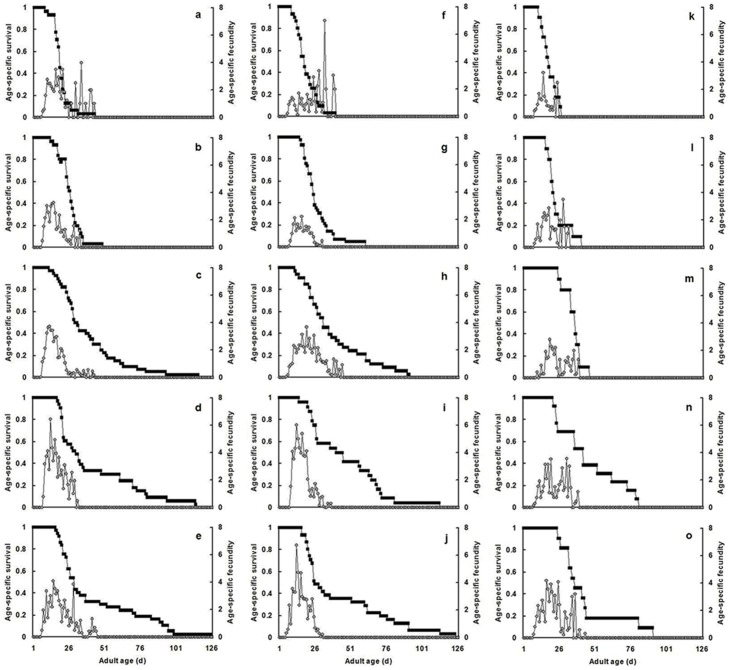
Relationship between age-specific survival (black) and age-specific fecundity (gray) for three *Adelphocoris* species under different levels of relative humidity (RH). (a), (b), (c), (d) and (e) are when *A. lineolatus* were exposed to 40%, 50%, 60%, 70% and 80% RH, respectively; similarly (f) to (j) are when *A. suturalis* were exposed to 40%–80% RH, and (k) to (o) are when *A. fasciaticollis* were exposed to 40%–80% RH.

For each of the *Adelphocoris* species, female and male adult longevity differed significantly among RH regimes (*A. lineolatus*: females, *F*_4,167_ = 7.71, *P*<0.001; males, *F*_4,167_ = 8.19, *P*<0.001; *A. suturalis*: females, *F*_4,50_ = 6.97, *P*<0.001; males, *F*_4,50_ = 11.12, *P*<0.001; and *A. fasciaticollis*: females, *F*_4,156_ = 9.08, *P*<0.001; males, *F*_4,156_ = 8.07, *P*<0.001). Adults of all three *Adelphocoris* spp. had the shortest longevity at 40% RH and generally lived the longest at 70% RH, with the exception of females of *A. lineolatus*, which lived longer at 80% RH. The fecundity among females of the three *Adelphocoris* species varied substantially among RH regimes (*A. lineolatus*: *F*_4,167_ = 6.68, *P*<0.001; *A. suturalis*: *F*_4,50_ = 12.57, *P*<0.001; and *A. fasciaticollis*: *F*_4,156_ = 24.28, *P*<0.001). The highest fecundity for *A. lineolatus* and *A. suturalis* occurred at 70% RH and for *A. fasciaticollis* at 80% RH; these levels were significantly higher than those at 40 and 50% RH (Table 1). The age-specific survival rates and fecundity also varied greatly with RH (Fig. 2). Females reared from egg hatch at low humidity (e.g., 40 and 50%) experienced sharply lower survival and fecundity than females reared at high humidity (e.g., 70 and 80%).

### Effect of humidity on life table parameters

The intrinsic rate of increase (*r_m_*), the net reproductive rate (*R_0_*), and the finite rate of increase (*λ*) increased with increasing RH, with only a slight decline in these rates for *A. lineolatus* and *A. suturalis* at 80% RH. The relationships between RH level and intrinsic rate of increase (*r_m_*), net reproductive rate (*R_0_*), and finite rate of increase (*λ*) for *A. lineolatus*, *A. suturalis* and *A. fasciaticollis* were described by a simple linear model (*P*<0.05 for all), but this was not the case for the mean generation time (*T*, all *P*>0.05) (Table 2).

**Table 2:** Effect of relative humidity on the life table parameters of three *Adelphocoris* species.

Species	Relative humidity (%)	Intrinsic rate of increase (*r_m_*)	Net reproductive rate (*R_0_*)	Mean generation time (*T*) (d)	Finite rate of increase (*λ*)
	40	0.0068	1.32	41.42	1.01
	50	0.0244	2.76	41.62	1.02
	60	0.0470	6.72	40.55	1.05
*Adelphocoris*	70	0.0609	13.34	42.53	1.06
*lineolatus*	80	0.0562	11.67	43.70	1.06
	Linear model	y = 0.14x-0.04	y = 31.26x-11.59	y = 5.46x+38.69	y = 0.14x+0.96
	*F*_1,3_	21.24	20.44	3.25	21.38
	R^2^	0.88	0.87	0.52	0.88
	*P*	0.0192	0.02024	0.1694	0.0190
					
	40	−0.0174	0.47	44.04	0.98
	50	0.0123	1.70	43.12	1.01
	60	0.0478	9.39	46.84	1.05
	70	0.0626	14.48	42.68	1.06
*Adelphocoris*	80	0.0606	13.32	42.74	1.06
*suturalis*	Linear model	y = 0.21x-0.09	y = 38.48x-15.21	y = -3.03x+45.70	y = 0.21x+0.91
	*F*_1,3_	22.36	21.72	0.25	22.94
	R^2^	0.88	0.88	0.08	0.88
	*P*	0.0179	0.0186	0.6539	0.0173
					
	40	−0.0138	0.55	43.07	0.99
	50	0.0174	2.20	45.41	1.02
	60	0.0333	5.18	49.38	1.03
*Adelphocoris*	70	0.0470	9.35	47.52	1.05
*fasciaticollis*	80	0.0568	15.79	48.59	1.06
	Linear model	y = 0.17x-0.07	y = 37.63x-15.96	y = 13.15x+38.91	y = 0.17x+0.92
	*F*_1,3_	54.36	50.66	5.81	59.85
	R^2^	0.95	0.94	0.66	0.95
	*P*	0.0052	0.0057	0.0950	0.0045

Means (± SEM) within a column followed by different letters are significantly different (*P*<0.05). Simple linear models show the relationship between relative humidity and life table parameters of three *Adelphocoris* species.

## Discussion

Relative humidity significantly affects immature survival and development and adult longevity and fecundity in a variety of insect species [21], [22], [23], [24], [25]. In this study, we confirmed that RH was a key determinant in the population growth of the economically important mirid bugs, *A. lineolatus*, *A. suturalis* and *A. fasciaticollis*.

In general, high humidity (i.e., 60, 70 and 80% RH) significantly increased egg and nymph survival of all three *Adelphocoris* species, whereas low humidity (i.e., 40 and 50% RH) had a detrimental effect on egg and nymph survival. These findings were consistent with the results of previous studies on these species [17], [26]. Ting [26] reported that egg hatch of *A. lineolatus* and *A. suturalis* was highest at a RH of 70% or higher at an ambient temperature of 25°C. Li et al. [17] also found that the egg hatch rate of *A. lineolatus* with high humidity (e.g., 80% RH) was significantly higher than with low humidity (e.g., 60% RH), when held at the same temperature. However, we found that the development times of eggs and nymphs of these three *Adelphocoris* species were not greatly affected by RH. This result was consistent with that in a previous study on *A. lineolatus* conducted with combinations of four temperatures and two levels of humidity [17]. In contrast to these results, studies have found strong effects of ambient humidity on the development rates of many insect species. For example, Han et al. [24] reported that low RH (20%) prolonged the development time and reduced the body mass of *Dendrolimus tabulaeformis* (Lepidoptera: Lasiocampidae) larvae when compared with 40%, 60% and 80% RH. Holemes et al. [27] reported that egg eclosion and adult emergence success of black soldier flies, *Hermetia illucens* (L.), increased with increasing RH, whereas the development time decreased with rising RH. However, the physiological mechanisms to explain the various responses of insects to different levels of humidity remain unclear. Based on previous studies [21], [22], [24], we inferred that RH greatly affected insect behavioral (e.g., feeding behavior) or physiological (e.g., water balance) traits, which in turn affected survival, development, and other parameters. The mechanisms and corresponding outcomes should receive more attention in further studies.

For each *Adelphocoris* species tested, both female and male adult longevity at high RH (i.e., 70% RH) were significantly greater than those in the lower RH range (i.e., 40% RH). Similarly, female fecundity was significantly higher at high RHs (i.e., 60, 70 and 80% RH) than fecundity at 40 and 50% RH. This result was consistent with the results of a previous study on *A. lineolatus* [17]. Similar trends due to increasing humidity were found for the intrinsic rate of increase (*r_m_*), the net reproductive rate (*R_0_*) and the finite rate of increase (*λ*) of each *Adelphocoris* species in this study and of *A. lineolatus* in another study [17]. The declines in several life table parameters of the mirid bugs at 80% RH may be caused by the negative effects from water drops that formed in glass vials at high humidity. However, this did not change the trend that populations of *Adelphocoris* spp. increased with higher humidity.

Populations of *Adelphocoris* generally increase rapidly after heavy rainfall, sometimes reaching outbreak levels [1], [2], [8], [14]. One striking example occurred in Anyang, Henan Province (36.10°N and 114.35°E) in 1954 when the average humidity was less than 60% in May. Only a small number of *A. fasciaticollis* eggs hatched at first, but a large number of eggs began to hatch when the humidity surpassed 60% after a heavy rain at the beginning of June. In 1955, the average humidity was less than 50% in May and June in this site, but a heavy rain caused an outbreak of *A. fasciaticollis* nymphs at the beginning of July [1]. Similar phenomena have been reported for *Adelphocoris* species in many other studies [3]. One possible explanation is that plants typically grow more rapidly after heavy rainfall, so sufficient suitable plant food is available to accelerate the insect population growth [3], [16], [17]. In this study, we demonstrated that an increase in RH after a heavy rainfall promoted directly the increase in population levels of *Adelphocoris* species and that RH may be a key factor associated with the outbreaks of *Adelphocoris* species observed after rainfall in China [1], [2], [8], [14], [15]. Rainfall with different characteristics could exert different effects on the survival of insects [8], [14]. The effects of rainfalls of different intensity, duration, or frequency on the population growth and seasonal dynamics of *Adelphocoris* species merit further study.

**Figure 3:**
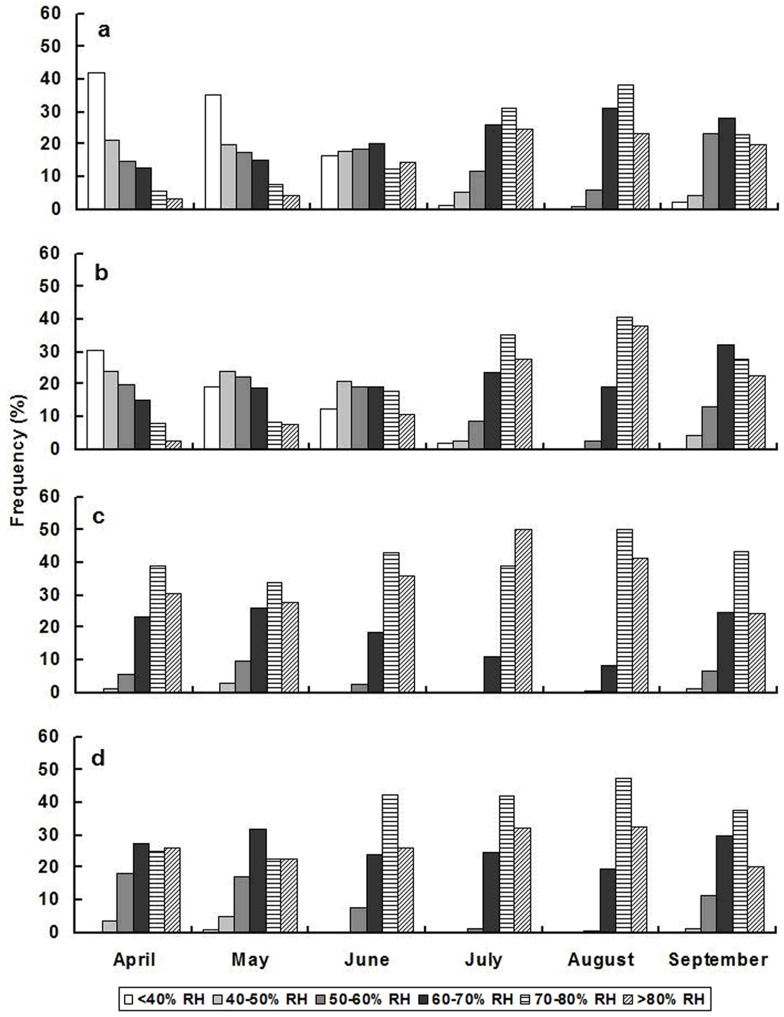
Frequency of relative humidity (RH) from April to September during 2001–2011 in the four main cotton growing locations in China. (a) Bazhou (Hebei Province, 39.06°N and 116.24°E); (b) Botou (Hebei Province, 38.04°N and 116.34°E); (c) Jingzhou (Hubei Province, 30.33°N and 112.23°E); and (d) Tianmen (Hubei Province, 60.39°N and 113.10°E). The sites a and b are located in the middle and northern parts of the Yellow River region, respectively, and the sites c and d are located in the Yangtze River region. In the early-season (i.e., from April to June), the RH is generally lower than 60% in Bazhou and Botou, but typically surpasses 60% RH in Jingzhou and Tianmen. During the summer season, the frequency of >70% RH is also higher in Jingzhou and Tianmen than the other two locations. In summary, the RH from April to September is always higher in the Yangtze River region than in the Yellow River region.

*Adelphocoris suturalis* is found mostly in the Yangtze River region and the southern part of the Yellow River region, whereas *A. lineolatus* and *A. fasciaticollis* are confined to the middle and northern parts of the Yellow River region [2]. In the spring (i.e., from April to June), the RH largely surpassed 60% in the Yangtze River region, but remained lower than 60% in the Yellow River region (Fig. 3). Such differences suggested that in the Yangtze River region the overwintering eggs of *A. suturalis* had higher hatch rates and their nymphs were more likely survive to adulthood, but that *A. lineolatus* and *A. fasciaticollis* were less likely to survive in the lower humidity of the Yellow River region. Therefore, we have a likely explanation for why *A. suturalis* typically reached high population levels on early season host plants and caused serious damage to cotton during the summer in the Yangtze River region, whereas the infestation levels of *A. lineolatus* and *A. fasciaticollis* remained relatively low throughout the year in the Yellow River region [2], [3].

The RH greatly affected the intrinsic rate of increase (*r_m_*) of each *Adelphocoris* species. The linear relationship between *r_m_* and RH had a higher slope (0.0021) for *A. suturalis* than for either *A. lineolatus* (0.0013) or *A. fasciaticollis* (0.0016). In particular, the *r_m_* for *A. suturalis* was higher than that for the other two species when the humidity increased from 60% RH to 80% RH, which indicated that high humidity more strongly affected the population growth of *A. suturalis* than the population growth of the other two species. This was consistent with the observations that *A. suturalis* usually had high population levels, which led to outbreaks in the high-humidity Yangtze River region [2], [3]. The more limited increase in the *r_m_* for *A. lineolatus* and *A. fasciaticollis* with rising humidity (from 50 to 70% RH) might indicate an ecological adaptation of these species to the low humidity in the drier Yellow River region and might be why these two species typically have higher population levels than *A. suturalis* when the species coexist within the Yellow River region [1], [26].

This study provides important data for understanding the population dynamics of three *Adelphocoris* species and for development of potential population dynamic models and pest forecasting at the regional scale.
